# Dynamic Covalent Polymer Networks of Silicone Elastomers via Organoborane Lewis‐Pairs

**DOI:** 10.1002/chem.202501595

**Published:** 2025-06-11

**Authors:** Edip Ajvazi, Verena Schinegger, Felix Bauer, Diana Drechsler, Patrick Rettenwander, Dominik Kaineder, Milan Kracalik, Oliver Brüggemann, Sabine Hild, Ingrid Graz, Uwe Monkowius, Ian Teasdale

**Affiliations:** ^1^ Institute of Polymer Chemistry Johannes Kepler University Linz Altenberger Straße 69 Linz 4040 Austria; ^2^ Institute of Polymer Science Johannes Kepler University Linz Altenberger Straße 69 Linz 4040 Austria; ^3^ Institute of Inorganic Chemistry Johannes Kepler University Linz Altenberger Straße 69 Linz 4040 Austria; ^4^ School of Education STEM Education Johannes Kepler University Linz Linz 4040 Austria

**Keywords:** covalent adaptive networks, dynamic covalent polymer networks, inorganic polymers, Lewis pairs, polydimethylsiloxane

## Abstract

Dynamic covalent polymer networks (DCPNs) have been developed in recent years to offer distinctive mechanical properties and shape responsiveness in covalently cured polymer materials, thereby bestowing them with desirable characteristics such as self‐healing and recyclability. To achieve these desirable properties, dynamic polymers incorporate reversible covalent bonds that enable shape morphing without causing irreversible chemical degradation of the network structure. Lewis pairs (LP), forming coordinate covalent bonds, sometimes referred to as dative covalent bonds, exhibit a broad range of bond dissociation rates and energies, making them inherently interesting for designing DCPNs. However, LPs have been relatively unexplored as polymer building blocks. Here, we present a straightforward approach to prepare LP‐based polymers based on archetypical nitrogen‐borane adducts with an organoborane‐functionalized polydimethylsiloxane combined with commercially available Lewis base (LB) polymers. The dynamic behavior of the organoborane‐functionalized system and LB polymers is investigated, demonstrating tunable reversibility in cured elastomers. The excess of LB facilitates rapid recapture and reformation of the covalent network, contributing to the system's reversible and self‐healing nature. Notably, the system allows easy mixing of different LB polymers, enabling the creation of tuning the system, and hence organoborane‐based LPs are demonstrated to be a promising building block for tunable DCPNs.

## Graphical abstract

DCPNs with reversible Lewis pair bonds are reported. Nitrogen‐borane adducts impart dynamic mechanical properties, such as self‐healing and recyclability, to polydimethylsiloxane elastomers.

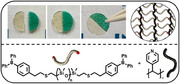

## 1 Introduction

Dynamic covalent polymer networks (DCPNs),^[^ ^1^ ^]^ also referred to as covalent adaptable networks (CANs),^[^ ^2^ ^]^ represent a key concept in current‐day polymer science to describe how dynamic reversible chemistry is utilized in cured polymer networks.^[^ ^3^ ^]^ Traditional network polymers, such as thermosets and elastomers, have strong covalent bonding, which is essential to their proven performance and immense industrial value. However, precisely this strong molecular bonding is a hindrance in terms of reprocessability and sustainability. Hence, introducing dynamic bonding into network polymers, while still retaining many of the beneficial properties of covalently cured polymers is used to enhance processability and recyclability as well as generate novel smart materials.^[^ ^4^ ^]^ The reversible crosslinks can be supramolecular based,^[^ ^5^ ^]^ utilizing noncovalent systems such as hydrogen bonding or host‐guest interactions or reversible molecular bonding such as metal–ligand coordinate bonding.^[^ ^6^ ^]^ Indeed, many dynamic covalent chemistries (DCC) have been incorporated into network polymers in recent years.^[^ ^1^, ^7^ ^]^ Examples of reversible covalent bonds used to prepare DCPNs include Diels‐Alder, oxime‐urethane exchange, and esters, coined vitrimers.^[^ ^2^ ^]^

**Figure 1:**
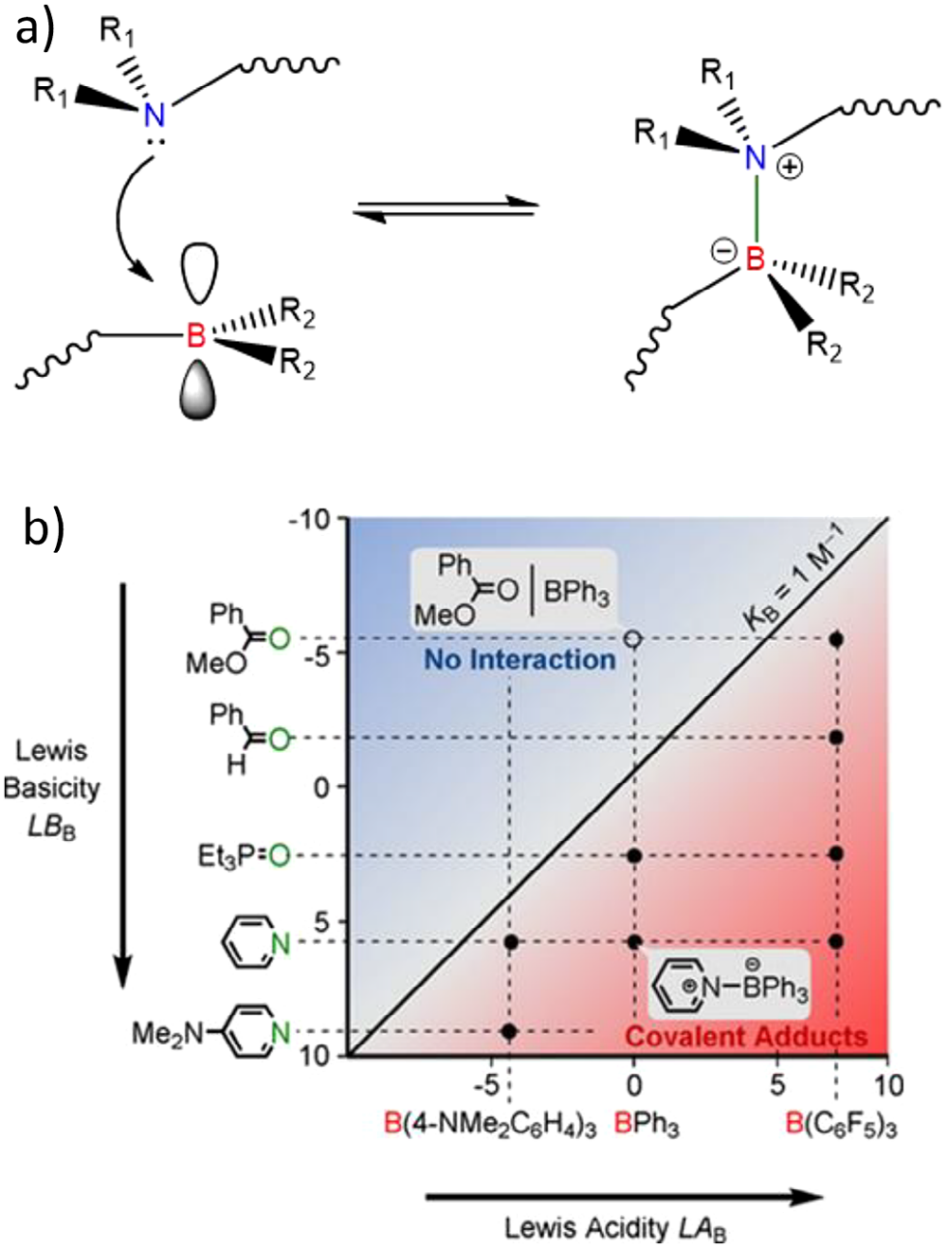
a) The Lewis pair platform used in this study. When attached to polymers (squiggle), the R_1_ and R_2_ moieties define the dynamic properties of the resulting covalent polymer. b) Graphical depiction of bond strength adapted from Ofial et al.^[^ ^8^ ^].^

Lewis pairs (LP), long known for their reversible nature, have not been given much attention in the recent rapid developments of DCPNs. In LP, the formed bond is a covalent bond, and the often added “dative” refers merely to the origin of the bonding electrons.^[^ ^9^ ^]^ Hence, at suitable bond strengths, they can also be classified as a DCC. There have been many attempts to quantify Lewis acidity and basicity in order to predict the strength of Lewis acid‐base interactions and the equilibrium conditions. However, there is no obvious structure‐property relationship which can describe the bonding energy and/or equilibrium with a single property scale for any combination of Lewis acid (LA) and Lewis base (LB). Such simple descriptions are usually limited to a specific group in LA and LB and cannot be generalized. Recently, however, Ofial and coworkers performed extensive thermodynamic and spectroscopic investigations to elucidate the equilibrium constants and bonding strength of the archetypal LP based on a nitrogen LB and a boron LA, which we utilized for the design in our studies. Importantly for designing DCPNs, the bond strength and the rate of dissociation can be easily tuned by R_1_ and R_2_ (Figure 1).^[^ ^10^ ^]^ This means that the response temperatures and kinetics of DCPNs constructed from these LPs can potentially be systematically tuned across a large range. Furthermore, the direct release and capture mechanism is relatively straightforward compared to many commonly used dynamic chemical reactions, such as transesterification and transamidation reactions.

Several groups have successfully incorporated boron‐nitrogen bonds into polymers,^[^ ^10^, ^11^ ^]^ including cleavable polymers.^[^ ^12^ ^]^ However, the use of LPs as dynamic covalent bonds in materials chemistry remains scarce. Brook was able to demonstrate that boronate‐functionalized PDMS could reversibly crosslink using Lewis pair complexes^[^ ^13^ ^]^ but these early studies did not systematically explore the mechanical tunability, recyclability, or self‐healing potential of these materials. Pioneering work from Jäkle et al. recently showed the proof‐of‐principle polymerizable aryl organoborane LAs combined with LB‐functionalized PDMS to provide transient networks.^[^ ^14^, ^15^ ^]^ The combination of accessible synthesis and high tunability makes such organoborane LPs excellent candidates for the preparation of DCPNs.^[^ ^14^, ^16^ ^]^ Meanwhile, Shaver et al. have developed frustrated Lewis pairs (FLPs), in which a sterically hindered LB has been used to prepare dynamic gels with a small‐molecule cross‐linker to prepare dynamic gels capable of self‐healing.^[^ ^17^, ^18^ ^]^ In this work we design a simple, readily tunable organoborane LP system through the functionalization of commercial PDMS with organoborane LAs and combining these with commercially available LB polymers in a robust, modular system. We study the stability of the organoborane system and the dynamic properties of the resulting cured polymers.

## 2 Results

### 2.1 Molecular Design and Small Molecule Models

**Figure 2:**
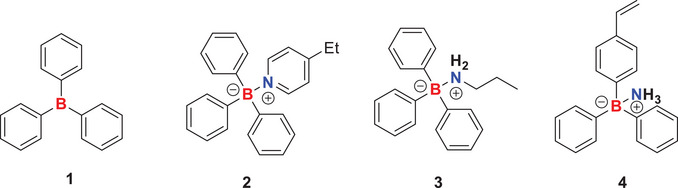
Chemical structure of the organoborane Lewis acids and their LP used in this study as small molecule models for the determination of basic properties.

**Figure 3:**
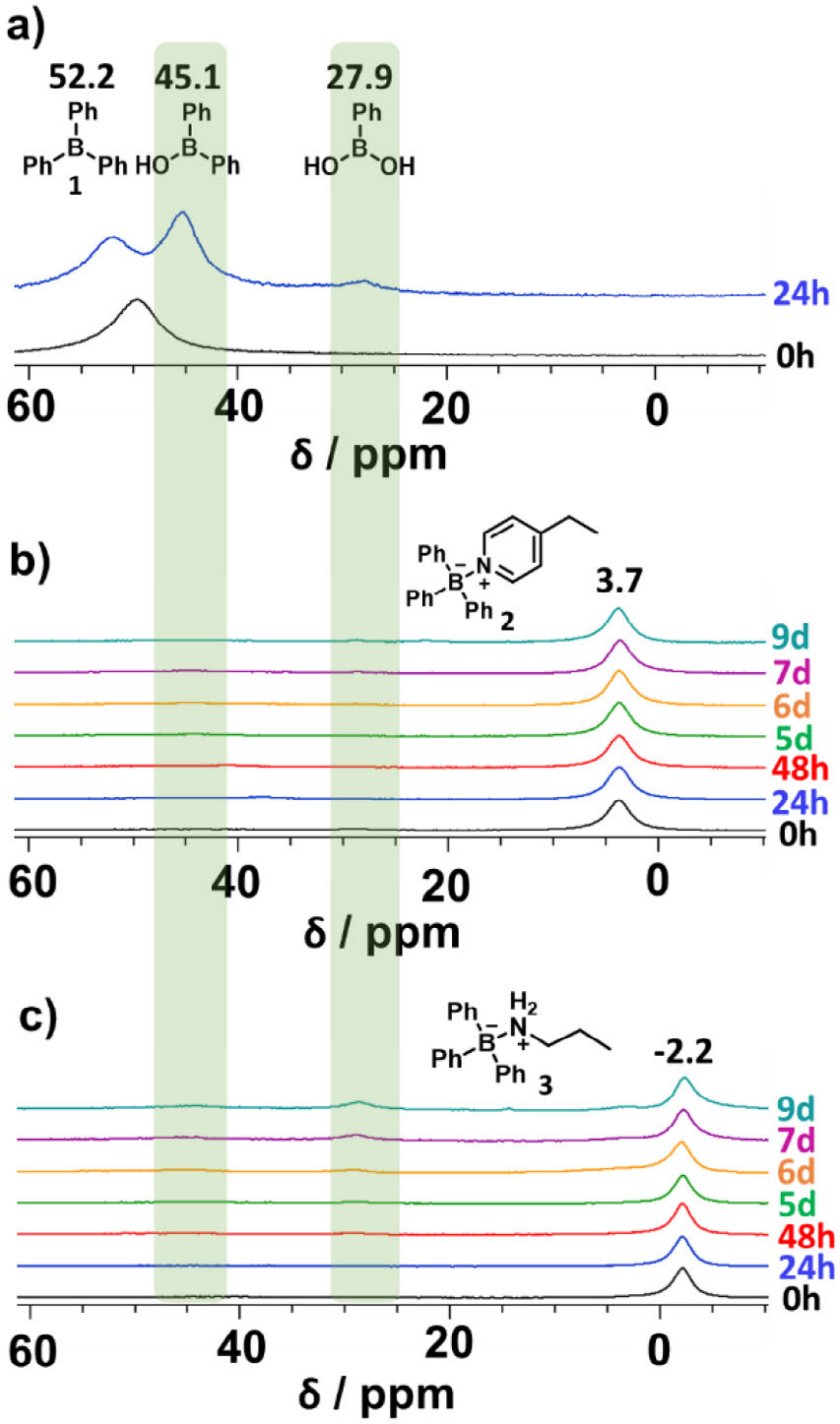
^11^B NMR stability study of a) triphenylborane (**1**), b) LP with ethylpyridine (**2**) and c) LP with propyl amine (**3**) in THF‐d_8_ under ambient conditions. While BPh_3_ is highly moisture sensitive, the LPs with pyridine can be handled on the benchtop and hence are suitable precursors for polymer materials.

An important concern with the application of organoborane chemistry for the preparation of functional polymer materials is their hydrolytic sensitivity. Hence, we first conducted an ^11^B NMR study with recrystallized BPh_3_ (**1**), ethylpyridine (**2**), and propyl amine (**3**) in THF‐d_8_ (Figure 2). When left open on the benchtop under ambient conditions, BPh_3_ (solid) rapidly hydrolyzed to Ph_2_B(OH) and further to PhB(OH)_2_ during the first 24 hours, visible by the arising peaks at 45.1 and 27.9 ppm, respectively (Figure 3a). Furthermore, the peak of pure BPh_3_ (0 hour, black) showed a slight downfield shift after 24 hours. This is always observable when a mixture of hydrolysis products and pure BPh_3_ is present, leading to a BPh_3_ peak shift difference of about 2 to 3 ppm. This behavior forbids polymerization and materials synthesis under air exposure and requires dry inert atmospheres for handling. However, we observed that the ethylpyridine adduct **2** had good hydrolytic stability, showing only the main adduct peak at 3.7 ppm for the 9 days of measuring time (Figure 3b). The conditions for the adduct stability study were the same as for the BPh_3_, namely, the study was conducted with the solid exposed to air under ambient conditions. The comparative study of the propyl amine adducts **3** exhibited in contrast to the ethylpyridine adduct less hydrolytic stability. Here, next to the adduct peak at ‐2.2 ppm, hydrolysis peaks indicating PhB(OH)_2_ species evolve after 7 and 9 days (Figure 3c). No Ph_2_B(OH) could be detected indicating that the second hydrolysis step is apparently accelerated in the presence of an amine base. This suggests that both binding strength and steric hindrance contribute to the observed stability, with ethylpyridine exhibiting superior hydrolytic resistance due to its stronger coordination and increased steric shielding against water.

Based on these results, we hypothesized that an excess of the LB would maintain the equilibrium on the side of the Lewis pair, and thus, in the subsequent materials, it should be possible to use this chemical building block for practical preparation of DCPNs without the need for extensive air‐free techniques. Indeed, similar pyridine‐organoborane LP compounds have been used commercially in antifouling coatings on ship hulls in protective hydrophobic polymer coatings.^[^ ^19^ ^]^ Shaver et al. recently reported the synthesis of a 4‐styryl‐diphenylboranes^[^ ^17^ ^]^ which we chose as a platform for subsequent LP polymers. We also observed that the amine adduct (**4**) of this species was also stabilized by the presence of an LB and hence could also be a useful Lewis acid for polymers. Indeed, this could be handled and stored on the benchtop for over one year (Figure SI 1b and 1c).

### 2.2 Polymer Synthesis

We prepared 4‐styryl‐diphenylborane ammoniate (**4**) from the Grignard derivative of 4‐bromostyrene and 2‐aminoethyl diphenylborinate by a modified literature procedure^[^ ^17^ ^]^ and the purity of the product and the successful reaction were confirmed by ^1^H and ^11^B NMR spectroscopy (Figure SI 1).

**Scheme 1:**
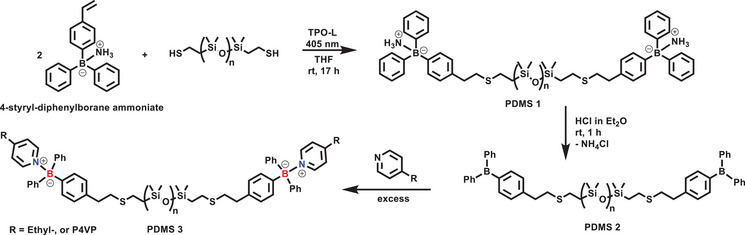
Synthesis of PDMS‐based dynamic covalent networks. Thiol‐ene addition forms **PDMS 1** followed by HCl treatment to yield **PDMS 2**. Complexation with Lewis bases (e.g., ethyl pyridine or P4VP) generates **PDMS 3**.

The styryl group was then coupled to an α,ω‐dithiol PDMS via a thiol‐ene addition reaction to obtain 4‐(diphenylborane‐ammoniate)phenethyl‐terminated **PDMS** **1** (Scheme 1). The reaction was carried out in the presence of ethyl(2,4,6‐trimethylbenzoyl)‐phenylphosphinate (TPO‐L) as a photoinitiator under 405 nm LED irradiation overnight. The used PDMS precursor had an average chain length of 70 repeat units and was synthesized in a two‐step process: first, commercially available divinyl‐terminated PDMS was functionalized via a thiolene‐addition reaction using thioacetate, followed by subsequent reduction by LiAlH₄ to yield the α,ω‐dithiol PDMS. Full conversion of the double bonds and thiol groups was confirmed by ¹H NMR spectroscopy (Figure SI 2). As with the model compounds, the organoborane α,ω‐chain end‐functionalized **PDMS 1** exhibited good stability and could be stored and handled on the open bench without noticeable hydrolysis (Figure SI 3).

**Figure 4:**
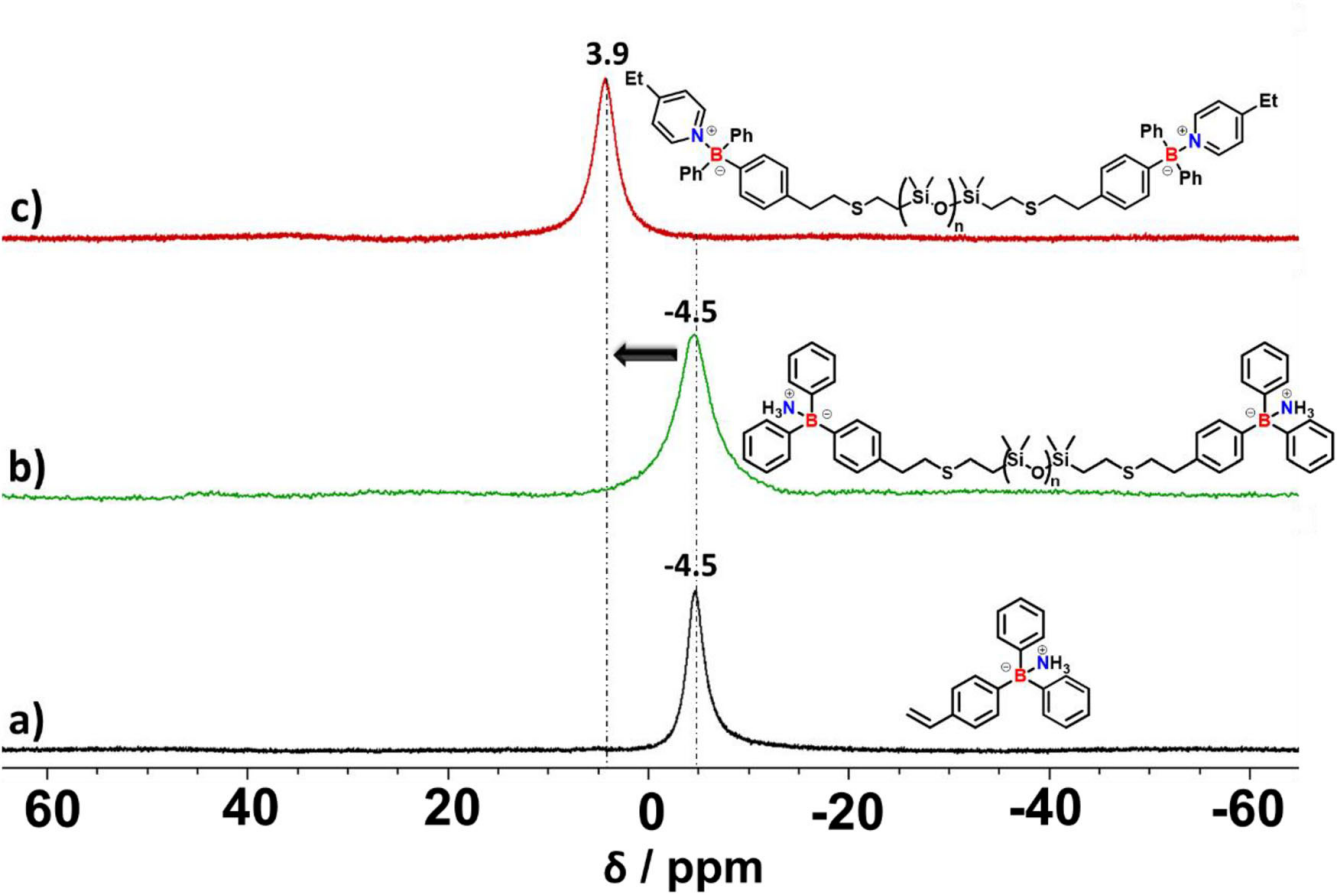
^11^B NMR spectra in THF‐d_8_ of different PDMS derivatives, confirming end‐functionalization. a) 4‐styryl‐diphenylborane ammoniate, b) PDMS functionalized with ammoniate‐protected borane (**PDMS 1**), and c) final Lewis pair complex with ethylpyridine (**PDMS 3**), showing characteristic chemical shifts.

The polymer was then washed with HCl in anhydrous diethyl ether before use to give the free arylborane LA **PDMS 2**, which was treated with an excess of pyridine to give the Lewis pair (**PDMS 3**). As can be seen in Figure 4, the peak in the ^11^B NMR spectra shifted downfield from ‐4.5 to 3.9 ppm, constituting the reference value for the DCPNs shown below. Ethyl pyridine models were used to mimic the electronic effects of the alkyl substitution in P4VP, although steric hindrance of neighboring groups could hinder the binding and is difficult to characterize in a complex cured polymer system.

### 2.3 Curing and Self‐healing Behavior

**Scheme 2:**
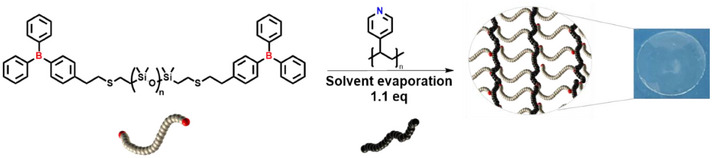
Curing of the dynamic covalent elastomer network.

The polymerization concept is shown in Scheme 2. Adding small amounts (3.9 wt% by mass, ∼1:1.1 eq. LA/LB) of P4VP as a dynamic crosslinker introduces a slight excess of Lewis basic (LB) pyridine relative to the Lewis acid (LA), which protects the Lewis acidic borane moiety against hydrolysis within an overall highly hydrophobic (96% wt% by mass) PDMS polymer network. The curing process relies on the formation of dynamic‐covalent boron‐nitrogen bonds, which allow the network to be both mechanically robust and reversibly crosslinked. The curing was carried out at room temperature under inert conditions without needing external catalysts or elevated temperatures, demonstrating the high reactivity of the organoborane‐based LPs. The gelation process was observed within seconds after mixing, leading to the formation of a viscoelastic network. The clear dominance of G′>G′′ across the frequency range (Figure SI 4a) indicates a robustly crosslinked, elastically dominated network. The amplitude sweep further supports this interpretation (Figure SI 4b).

**Figure 5:**
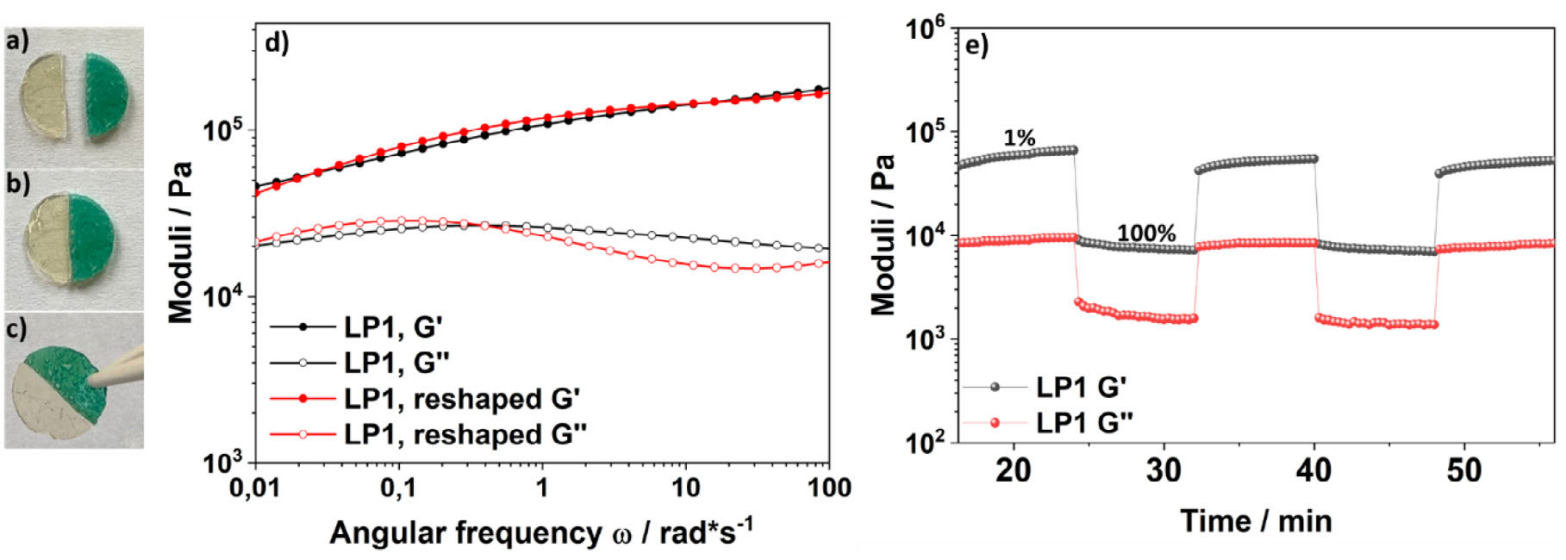
Self‐healing and rheological behavior of the dynamic covalent elastomer. a)–c) Macroscopic self‐healing test showing the rejoining of a cut sample over time. d) Frequency‐sweep measurements before and after reformation, demonstrating the recovery of the moduli. e) Step‐strain rheology test showing the reversible disruption and reformation of the network under alternating strain amplitudes over five cycles (1% and 100%).

The self‐healing capability of the network was investigated by macroscopic cut‐healing tests and rheological recovery experiments. The samples were cut into two separate pieces and pressed together without any external force or solvent (Figure 5a‐c). The recovery over time of the viscoelastic properties was evaluated, and after three days, the material exhibited nearly complete reformation, as determined by frequency‐sweep rheology, and confirmed that the reshaped material maintained nearly identical viscoelastic properties to the original cured polymer, underscoring the robustness of the reversible crosslinking system (Figure 5d).

To further analyze the self‐healing process, cyclic step‐strain rheology experiments were performed, in which the sample was subjected to alternating shear deformation cycles both within and beyond the linear viscoelastic regime (LVE), previously determined by amplitude sweeps (Figure SI 5). Upon entering the high‐strain phase (100%), both dynamic moduli G′ and G′′ dropped significantly and continued to decrease moderately throughout the deformation interval, indicating a transition beyond the LVE and progressive network disruption. When the strain was reduced back to 1%, the moduli immediately increased to higher values and further regenerated with each subsequent low‐strain step. This suggests that while most dynamic boron‐nitrogen crosslinks rapidly reform, a small fraction requires additional time to fully reestablish equilibrium. Over successive cycles, both G′ and G′′ stabilized at a reproducible level, confirming an adaptive, reversible crosslinking network. Long‐term recovery measurements (Figure SI 6) showed that after ∼13 hours, the modulus returned to near‐initial levels, demonstrating complete restoration of mechanical integrity. We postulate this efficient healing behavior to the presence of excess LB groups, which likely promote rapid re‐coordination and network reformation upon contact.

### 2.4 Tailoring Mechanical Properties

A series of elastomeric materials was then prepared based on an organoborane‐functionalized siloxane (**PDMS 2**) crosslinked with two commercially available LB components, namely the previously used P4VP and the primary amine‐functionalized PDMS (AMS162, 7% NH₂ groups). The mechanical properties of these materials are governed by a combination of backbone structure, molecular weight, and functional group density, which collectively influence the crosslinking density and network architecture. P4VP features a rigid, carbon‐based backbone with a high functional group density, as each repeating unit carries a pyridine group. This results in a highly crosslinked network that enhances mechanical strength but may limit polymer chain mobility. In contrast, AMS162 contains only 7% amine groups, leading to fewer crosslinking points. However, its flexible PDMS backbone provides high stretchability and improves network mobility. The combination of these two components enables the formation of a tunable network, balancing stiffness and adaptability. To identify an optimal balance between mechanical integrity and network dynamics, various formulations (Table SI 1) with different LB ratios were tested. Through an iterative optimization process, a formulation with a 10% excess of LB was found to provide the best compromise between rigidity and adaptability. In addition to improving mechanical properties, the excess of LB visually enhanced the network's resistance to dissolution. While formulations with a strict 1:1 stoichiometry readily dissolved upon exposure to an excess of solvent, the introduction of a 10% excess of LB reduced solubility. Although this observation is based on empirical comparison, it suggests that additional LB coordination contributes to a more stabilized, yet still dynamic, network structure.

**Figure 6:**
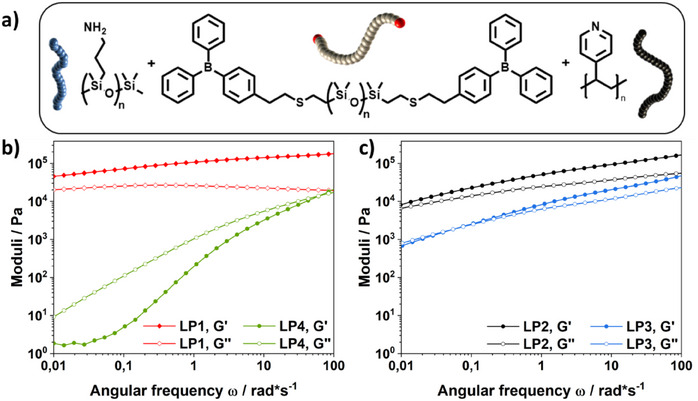
a) Molecular components used in the formation of the DCPNs. b) Frequency sweep analysis of LP1 and LP4. Storage modulus and loss modulus are plotted as a function of angular frequency, highlighting the strong elastic response of LP1 and the viscoelastic behavior with pronounced frequency dependence observed for LP4. c) The moduli of LP2 and LP3 fall between those of LP1 and LP4. Their frequency‐dependent increase in both G′ and G″ shows tunable viscoelastic properties and highlights distinct network dynamics.

The mechanical properties of the developed polymer networks were analyzed using frequency‐sweep measurements to investigate the effects of backbone structure, functional group density, and mixing ratio on their viscoelastic behavior. The four formulations (LP1, LP2, LP3, LP4) differ in their proportions of poly(4‐vinylpyridine) (P4VP) and amine‐functionalized PDMS (AMS162, 7% NH₂ groups), which directly influence their rheological characteristics. LP1, containing only P4VP as an LB (1.1 eq P4VP, 0 eq AMS), exhibits the highest storage modulus (G′) across the entire frequency range. The observed frequency sweep is phenomenologically consistent with the expected response of crosslinked polymer networks, supporting the previously described elastic‐dominated behavior. This pronounced mechanical strength is attributed to the high crosslinking density resulting from the functional groups of P4VP, as well as the increased entanglements and restricted mobility associated with its high molar mass. In contrast, LP4, which contains only AMS162 (0 eq P4VP, 1.1 eq AMS), displays a significantly lower G′ with a more pronounced increase at higher frequencies. This behavior suggests a lower crosslinking density and a more pronounced viscoelastic character. The comparison between G′ and G′′ reveals that the network is more influenced by dissipative processes, which is attributed to the increased molecular mobility imparted by the flexible PDMS backbone (Figure 6a). The LP2 formulation (0.55:0.55 eq P4VP/AMS) demonstrates a well‐balanced combination of both properties. While G´ remains higher than in LP4, the material still exhibits a certain degree of network mobility. This balance confirms that combining P4VP and AMS enables a tunable interplay between structural integrity and flexibility, which is reflected in the relatively high G′′ values, indicating enhanced molecular mobility. LP3 (0.35:0.75 eq P4VP/AMS) exhibits intermediate mechanical properties between LP4 and LP2, but with a more pronounced frequency dependence of G′, indicating a more dynamic network. The slightly increased viscous contribution compared to LP2 suggests that a higher AMS content further enhances network mobility (Figure 6b). Overall, the frequency sweep measurements confirm that P4VP significantly contributes to the mechanical stability of the network, while AMS enhances elasticity and molecular mobility. To further support these observations, the complex viscosity (η*) of all formulations was analyzed, resulting in the highest η* values for LP1 in the lower frequency range (Figure SI 7b), while LP4 shows consistently low viscosity across all frequencies, indicative of a weakly structured and easily deformable network. The formulations LP2 and LP3 exhibit again an intermediate behavior, demonstrating the tunability of the system. Stress relaxation experiments (Figure SI 8) further confirm the trends observed in the frequency sweep measurements, showing that LP1 exhibits the slowest relaxation dynamics due to its high crosslink density, while LP4 relaxes rapidly, indicating a highly dynamic and weakly crosslinked network. The intermediate formulations LP2 and LP3 display relaxation behavior consistent with their balance between elasticity and dynamic adaptability.

**Figure 7:**
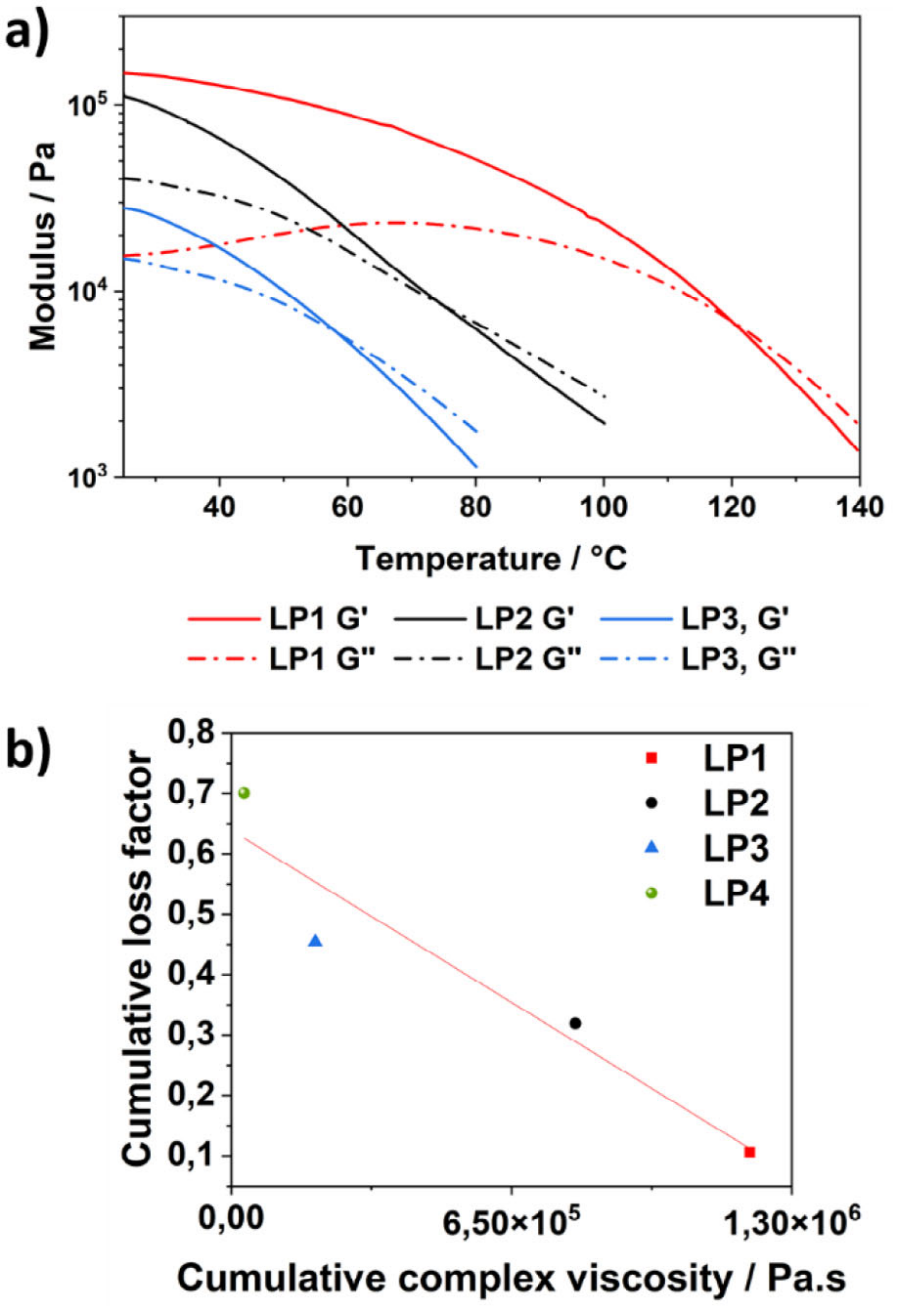
a) Temperature‐dependent storage and loss moduli of LP1–LP3, showing thermally induced softening due to dynamic bond exchange. b) Correlation between cumulative loss factor (CLF) and cumulative complex viscosity (CCV) (η∗) for LP1–LP4, highlighting the balance between structural integrity and damping capacity across the dynamic polymer networks.

The incorporation of dynamic bonding into polymers has raised interest in their potential use as energy‐dissipating materials.^[^ ^20^ ^]^ To evaluate this, the thermal viscoelastic behavior of the developed polymer networks was investigated by temperature‐sweep rheology (Figure 7a, SI 9). All formulations exhibit a temperature‐dependent softening of the network, with decreasing storage and loss moduli. LP1, which only contains P4VP as a crosslinking base, exhibits the highest storage modulus across the entire temperature range and maintains lower energy dissipation compared to the other formulations. In contrast, LP4, which incorporates only AMS, shows a pronounced modulus decrease and early crossover to a viscous‐dominated regime (G′′>G′), reflecting reduced network persistence. The mixed systems LP2 and LP3 fall between these extremes, demonstrating the tunability of the network structure. To better compare the interplay between energy dissipation and structural integrity, the cumulative loss factor (CLF) was plotted against the cumulative complex viscosity (CCV). The CLF quantifies the total energy dissipated across the measured frequency range, while the cumulative storage factor (CSF) reflects the overall ability of the material to store elastic energy. Both parameters were calculated using an integral‐based approach previously developed for assessing network stability in polymer nanocomposites.^[^ ^21^ ^]^ Figure 7b shows the correlation between CLF and CCV, offering a view of the damping capacity versus structural persistence. LP1 combines high viscosity with low CLF, confirming its predominantly elastic and robust network architecture. In contrast, LP4 shows low viscosity but a high CLF, indicative of a more dissipative yet less structurally strong network. LP2 and LP3 exhibit intermediate behavior, allowing viscoelastic fine‐tuning based on formulation. The corresponding CSF data are shown in the Supporting Information (Figure SI 10) and support these trends by highlighting the elastic energy retention across the frequency spectrum. These cumulative results are in line with the temperature‐dependent rheological profiles (Figure 7a) and reinforce the structure‐property relationships of the dynamic Lewis pair networks. Additional analysis of the temperature‐dependent loss factor tanδ (Figure SI 11) supports these findings. LP3, for example, maintains tanδ values near one over a wide temperature and frequency range, suggesting suitability for damping applications. Notably, the key viscoelastic responses of all networks fall within the range relevant for human motion and vibration frequencies 0.1–50 Hz (0.63–314 rad/s),^[^ ^22^ ^]^ highlighting their potential use in wearable impact protection or vibration‐damping systems.

**Figure 8:**
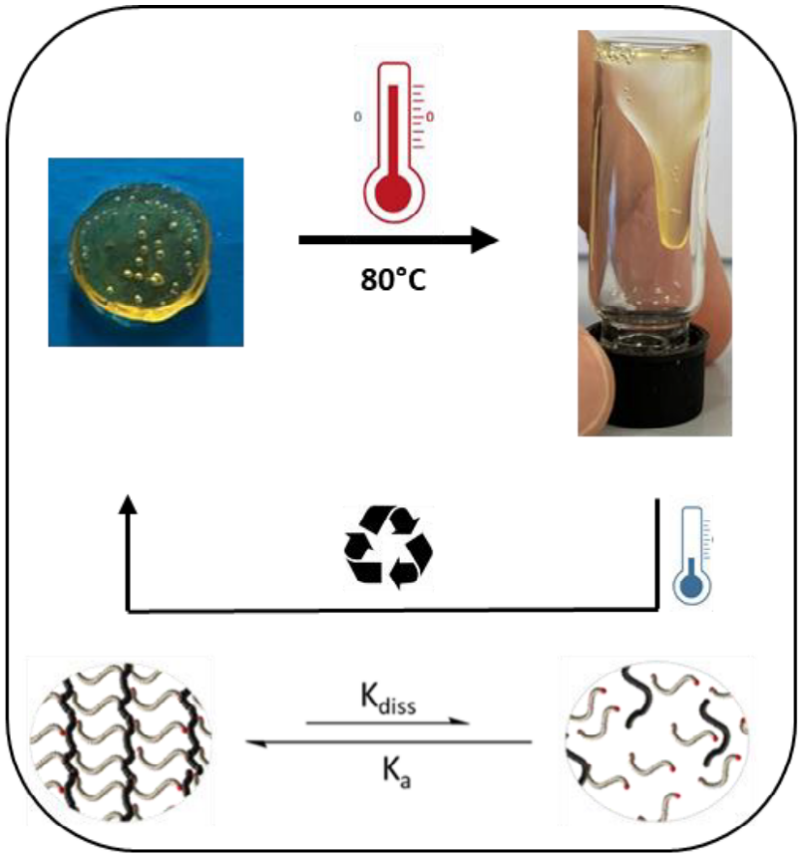
Thermal reversibility and recyclability of LP5. Upon heating, the crosslinked network undergoes dissociation, transitioning into a flowable state. Cooling restores the structured network, demonstrating the dynamic and reversible nature of the system.

Beyond their damping properties, these polymer networks were further evaluated for their thermal recyclability. While CANs are widely explored for polymer recycling, boron‐containing systems present unique challenges due to their susceptibility to oxidative degradation. To address this, we systematically investigated the impact of LB excess on thermal stability and recyclability. A key limitation of boron‐based polymer networks is their sensitivity to oxidation and hydrolysis, which can compromise recyclability under thermal processing. Our results indicate that increasing the LB excess significantly improves hydrolytic stability by stabilizing the boron centers and preventing their uncontrolled degradation. This effect is particularly evident in LP1, which, with a 10% excess of LB, already demonstrates improved stability but still exhibits a notable decrease in G′ and G″ after heating. To further enhance thermal recyclability, we developed LP5, a formulation with a substantially higher excess of LB. In contrast to LP1, which experiences a decrease in mechanical properties after thermal cycling, LP5 retains a much higher degree of mechanical stability, indicating that an increased LB concentration further reinforces the network against oxidative and hydrolytic degradation. This comparison suggests that while a moderate excess of LB provides some stabilization, it is not sufficient to fully prevent network degradation upon heating. The superior performance of LP5 demonstrates that a higher LB concentration more effectively shields boron centers, thereby maintaining crosslinking integrity and enhancing recyclability (Figure SI 12 and 13). This thermally induced dissociation‐reassociation behavior is schematically illustrated in Figure 8, highlighting the reversible transition between a crosslinked and a flowable state in LP5, which underpins its recyclability and dynamic network integrity.

## 3 Conclusion

In this study, we report the synthesis of a novel organoborane‐capped polydimethylsiloxane that can be used to prepare DCPNs with fully reversible and repeatable self‐healing properties. Our findings suggest that the combination of the selected LB, the stoichiometry of the system, and the hydrophobic siloxane polymer matrix enables the preparation of robust DCPN systems that can be handled and stored under ambient conditions. Furthermore, the material properties can be readily tailored by judicious choice of the Lewis pair combination. This advancement broadens the scope of organoborane chemistry in material science. Future work should explore additional structural modifications of the Lewis pair and adaptation to other polymer systems, with the overarching goal of developing fine‐tuned recyclable covalently cured polymers.

## 4 Materials and Methods

All reactions, unless otherwise stated, were carried out under inert conditions using an argon atmosphere, either with Schlenk techniques or inside an MBraun UNILab LMF glovebox. Chemicals were purchased from various commercial suppliers and used as received unless otherwise specified. Vinyl‐terminated polydimethylsiloxane (DMS V21, M ≈ 5200 g/mol) and amine‐functionalized PDMS (AMS 162, M ≈ 5000 g/mol) were obtained from Gelest and the approximate molecular weight was determined by ¹H NMR spectroscopy end‐group analysis. Additional reagents included ammonium chloride (VWR), 2‐aminoethyl diphenylborinate (BLD), 4‐bromostyrene (BLD), 4‐ethylpyridine (Sigma‐Aldrich), ethyl(2,4,6‐trimethylbenzoyl)‐phenylphosphinate (TPO‐L, Fluorochem), triphenylborane (ABCR), hydrogen chloride in diethyl ether (1 M) (VWR), deuterated solvents (Eurisotop), 1,2‐dibromoethane (Sigma‐Aldrich), and n‐heptane (VWR). Photochemical reactions were performed in a Wanhao Boxman‐1 UV light chamber at 405 nm. NMR spectra were recorded on a Bruker Avance 300 spectrometer at 300 MHz for ¹H NMR, 121 MHz for ¹¹B NMR. Quartz NMR tubes were used for ¹¹B NMR measurements. All spectra were referenced to the residual peak of the respective deuterated solvents.

Rheological characterization of the elastomer samples was performed using an Anton Paar MCR 502 rheometer equipped with a plate‐plate geometry. The elastomer samples were prepared with a diameter of 10 mm and a thickness of 1–2 mm, ensuring proper contact with the measurement suitable for the installed plate‐plate geometry of a 10 mm diameter. To determine the linear viscoelastic region (LVE), amplitude sweep tests were conducted at a constant angular frequency of 10 rad/s, with strain values ranging from 1 to 100%. Based on the identified LVE range, a dynamic frequency sweep was performed at a fixed strain within the linear region (γ = 1%), covering an angular frequency range from 0.01 to 100 rad/s. Temperature‐dependent rheological behavior was analyzed using temperature sweeps at a constant strain of 10% and an angular frequency of 10 rad/s, applying a heating rate of 1.5 °C per minute. To assess the network recovery behavior after mechanical disruption, cyclic step‐strain rheology experiments were performed at an angular frequency of 62.8 rad/s. Additionally, stress relaxation tests were performed exemplarily for dedicated samples at room temperature. To remain within the linear viscoelastic range (LVE), a strain of 1% was used uniformly.

### 4.1 ¹¹B NMR Study of Triphenylborane (BPh₃)

#### 4.1.1 Recrystallization and Storage

For the ¹¹B NMR studies, commercially obtained triphenylborane was recrystallized from diethyl ether and stored under an argon atmosphere inside a glovebox.

#### 4.1.2 BPh₃ Stability Study

Triphenylborane (15 mg, 0.061 mmol) was weighed into an Eppendorf vial and left open on the benchtop under ambient conditions. The sample was measured after 24 hours in 0.6 mL of THF‐d₈.

#### 4.1.3 Adduct Formation Studies

Triphenylborane (15 mg, 0.062 mmol, 1 eq.) was dissolved in 0.6 mL of THF‐d₈ and transferred to an NMR tube sealed with a septum cap. An LB (2 eq.) was then added using a microliter glass syringe, and the NMR tube was gently shaken for a few seconds before measurement. After the initial NMR measurement, the sample was transferred to a vial, and the solvent was removed by rotary evaporation. The remaining solid was left open on the benchtop until the next measurement. For subsequent measurements, the same sample was redissolved, measured, isolated again, and exposed to ambient conditions before the next analysis.

### 4.2 Synthesis

#### 4.2.1 Synthesis of 4‐Styryl‐Diphenylborane Ammoniate

The synthesis was adapted from a previously reported procedure.^[^ ^17^ ^]^ 2‐Aminoethyl diphenylborinate (4.0 g, 18 mmol) and magnesium turnings (1.1 g, 47 mmol) were suspended in anhydrous tetrahydrofuran (THF, 120 mL) under an inert atmosphere. 1,2‐Dibromoethane (0.1 mL) was added as an activator, and the mixture was stirred at room temperature for 30 minutes. Under exclusion of light, an initial aliquot of 4‐bromostyrene (0.56 g, 0.40 mL, 0.31 mmol) was added dropwise, and stirring was continued at RT for 30 minutes, until the reaction mixture developed a green color. The solution was then cooled in an ice bath, and an additional portion of 4‐bromostyrene (5.0 g, 3.6 mL, 28 mmol) was added dropwise over 20 minutes. The reaction was maintained at this temperature for 2 hours, followed by stirring at RT for an additional 2 hours. To quench the reaction, a saturated aqueous NH₄Cl solution (60 mL) was added, leading to a white color change. The mixture was stirred for 30 minutes, and the aqueous phase was separated and extracted with diethyl ether (3 × 20 mL). The combined organic layers were dried over anhydrous magnesium sulfate, and the solvent was removed under reduced pressure using a rotary evaporator. The resulting yellow oil was precipitated in n‐heptane (60 mL), yielding a white solid, which was isolated by suction filtration. The crude product was further purified by recrystallization from toluene, affording the final compound as a purified white solid (2.45 g, 8.6 mmol).

Yield: 69%; ¹H NMR (300 MHz, DMSO‐d₆, δ): 7.25–6.97 (m, 14H), 6.65 (dd, 1H), 5.68 (d, 1H), 5.60 (s, 3H), 5.09 (d, 1H) ppm; ¹¹B NMR (96 MHz, DMSO‐d₆, δ): ‐5.0 ppm.

#### 4.2.2 α,ω‐Dithiol‐terminated Polydimethylsiloxane (α,ω‐Dithiol‐PDMS)

The synthesis was adapted from the literature.^[^ ^23^ ^]^ Vinyl‐terminated polydimethylsiloxane (DMS V21, 20 g, 3.8 mmol) was dissolved in chloroform (20 mL) containing thioacetic acid (2 equiv per vinyl group) and TPO‐L (0.02 equiv) as a photoinitiator. The reaction mixture was degassed under vacuum and subsequently irradiated with UV light overnight. After completion, the solvent was removed under reduced pressure using a rotary evaporator, and the residue was diluted with diethyl ether. The solution was neutralized with a saturated aqueous NaHCO₃ solution, followed by two washes with deionized water. The organic phase was dried over anhydrous MgSO₄, and the solvent was removed under vacuum to yield PDMS‐(SAc)_2_ as a colorless viscous liquid.

Yield: 95%; ¹H NMR (300 MHz, CDCl₃, δ): (‐0.22)‐0.07 (m, 480H), 0.76–0.89 (m, 4H), 1.16–1.31 (m, 4H), 2.20–2.30 (s, 3H), 2.80–2.90 (m, 4 H) ppm.

To a degassed solution of the thioacetate‐functionalized PDMS‐(SAc)_2_ (10.0 g) in dry diethyl ether (10 mL), a dispersion of lithium aluminum hydride (LiAlH₄, 1.1 equiv per thioacetate group) in dry diethyl ether (60 mL) was added slowly under an argon atmosphere at 0 °C. The reaction mixture was stirred at 0 °C for 30 minutes, followed by stirring at room temperature for an additional 5 hours. The reaction was quenched by slow addition of 10% acetic acid, and the phases were separated. The organic layer was washed with a saturated aqueous NaHCO₃ solution, dried over anhydrous MgSO₄, and concentrated under reduced pressure to afford PDMS‐(SH)_2_ as a viscous liquid.

Yield: 86%; ¹H NMR (300 MHz, CDCl₃, δ): (‐0.22)‐0.05 (m, 480H), 0.86–0.98 (m, 4H), 1.36–1.47 (m, 2H), 2.29 (s, 4H), 2.32 (s, 0. H), 2.47–2.64 (m, 0.11 H) ppm.

#### 4.2.3 Synthesis of 4‐(Diphenylborane)phenethyl‐terminated PDMS (PDMS 1)

4‐Styryl diphenylborane (0.67 g, 2.4 mmol) was dissolved in 100 mL anhydrous tetrahydrofuran. α,ω‐Dithiol‐functionalized polydimethylsiloxane (PDMS‐(SH)_2_, 5.6 g, 1.1 mmol) was degassed for 20 minutes before adding TPO‐L (2.5wt%) as a photoinitiator. The reaction mixture was stirred at room temperature for 17 hours under an inert atmosphere and irradiated in a UV chamber at 405 nm. After completion, the product was purified by washing with n‐heptane and centrifugation. The solvents were removed under reduced pressure using a rotary evaporator, yielding a yellow sticky mass.

Yield: 61%; ¹H NMR (300 MHz, CDCl₃, δ): (‐0.30)‐0.22 (m, 480H), 0.72–0.90 (m, 4H), 1.98–2.25 (m, 4H), 2.45–2.60 (m, 4H), 2.63–2.82 (s, 4H), 3.77–4.10 (br, 6H), 7.25–6.86 (m, 28H) ppm; ¹¹B NMR (96 MHz, THF‐d_8_, δ): ‐4.5 ppm.

#### 4.2.4 Synthesis of 4‐(Diphenylborane)phenethyl‐terminated PDMS (PDMS 2)

The deprotection of **PDMS 2** was performed in analogy to the literature.^[^ ^17^ ^]^ All operations were carried out under an inert atmosphere inside a glovebox. 4‐(Diphenylborane‐ammoniate)‐phenethyl‐terminated PDMS (2.5 g, 0.40 mmol) was dissolved in anhydrous diethyl ether (14 mL). A solution of hydrogen chloride in diethyl ether (1 M, 2 mL) was added dropwise, and the reaction mixture was stirred at room temperature for 1 hour. The resulting white precipitate was removed by filtration. The solvent and unreacted hydrogen chloride were removed under ambient pressure to afford the product as a yellow oil.

For the model compound **PDMS 3**, 4‐ethylpyridine (1.1 equiv, relative to the boron centers in **PDMS 2**) was added to the reaction mixture, simulating the conditions used in the elastomer formulation.

Yield: 84%; ¹H NMR (300 MHz, THF‐d_8_, δ):): (‐0.30)–0.26 (m, 480H), 0.83–1.03 (m, 4H), 1.89–2.66 (m, 4H), 2.81–2.99 (m, 4H), 2.63‐.2.82 (s, 4H), 3.7 (br, 4H), 1.37–1.53 (m, 6H), 6.96–7.20 (m, 8H), 7.25–6.86 (m, 20H) ppm; ¹¹B NMR (96 MHz, THF‐d_8_, δ): 3.8 ppm.

#### 4.2.5 General Synthesis of Crosslinked Elastomers

In short, based on LP2 as an example, 300 mg of **PDMS 2** (from a 0.5 g/L stock solution in DCM) was mixed with 6 mg of P4VP (from a 0.1 g/L stock solution in DCM) and 69.5 mg of AMS162 in a glass vial in a glovebox. The components were stirred, leading to an immediate increase in viscosity, indicating the formation of the network structure. The solvent was gradually evaporated under inert conditions, and the resulting viscous mixture was transferred into a Teflon mold to shape the elastomer. The sample was allowed to stand for at least three days before any measurements were performed to ensure full equilibration of the dynamic network. The final material appeared as a light‐yellow, transparent elastomer.

## Author Contributions

*DI Edip Ajvazi*, writing‐original draft, investigation, methodology, visualization, data curation, conceptualization and formal analysis. *DI Verena Schinegger*, writing‐original draft, investigation, methodology. *Felix Bauer, B.Sc*., investigation, data curation. *DI Patrick Rettenwander*, investigation. *DI Diana Drechsler*, investigation. *DI Dominik Kaineder*, validation. *Assoc. Univ.‐Prof. Dr. Milan Kracalik*, validation, supervision. *Univ.‐Prof. Dr. Oliver Brüggemann*, review. *Univ.‐Prof. Dr. Sabine Hild*, review. *Assoc. Univ.‐Prof. Dr. Ingrid Graz*, conceptualization, funding acquisition. Assoc. Univ.‐Prof. Dr. Uwe Monkowius*, conceptualization, supervision, funding acquisition writing‐review and editing. Assoc. Univ.‐Prof. Dr. Ian Teasdale*, conceptualization, supervision, funding acquisition, writing‐review and editing

## Conflict of Interests

The authors declare no conflict of interest

## Supporting information



Supporting Information

## Data Availability

The data that support the findings of this study are available in the supplementary material of this article.
