# Supplementary material for: Dynamic Covalent Polymer Networks of Silicone Elastomers via Organoborane Lewis‐Pairs
Source: Chemistry. 2025 Jun 11;31(39):e202501595. doi: 10.1002/chem.202501595 (PMC12258663; doi:10.1002/chem.202501595)
Supplement: Supplementary file 1 — Supporting Information [file CHEM-31-e202501595-s001.docx]

**Supporting Information**

**Dynamic Covalent Polymer Networks of Silicone Elastomers via Organoborane Lewis-Pairs**

*Edip Ajvazi^1^, Verena Schinegger^1,4^, Felix Bauer^1,2^, Diana Drechsler^1^, Patrick Rettenwander^1^, Dominik Kaineder^2^, Milan Kracalik^2^, Oliver Brüggemann^1^, Sabine Hild^2^, Ingrid Graz^4^, Uwe Monkowius^3,4^* and Ian Teasdale^1^**

^1^ Institute of Polymer Chemistry, Johannes Kepler University Linz, Altenberger Straße 69, 4040 Linz, Austria

^2^ Institute of Polymer Science, Johannes Kepler University Linz, Altenberger Straße 69, 4040 Linz, Austria

^3^ Institute of Inorganic Chemistry, Johannes Kepler University Linz, Altenberger Straße 69, 4040 Linz, Austria

^4^ School of Education, STEM Education, Johannes Kepler University Linz, 4040 Linz, Austria.

Keywords: Covalent adaptive networks, Dynamic covalent polymer networks, Lewis pairs, polydimethylsiloxane. Inorganic polymers


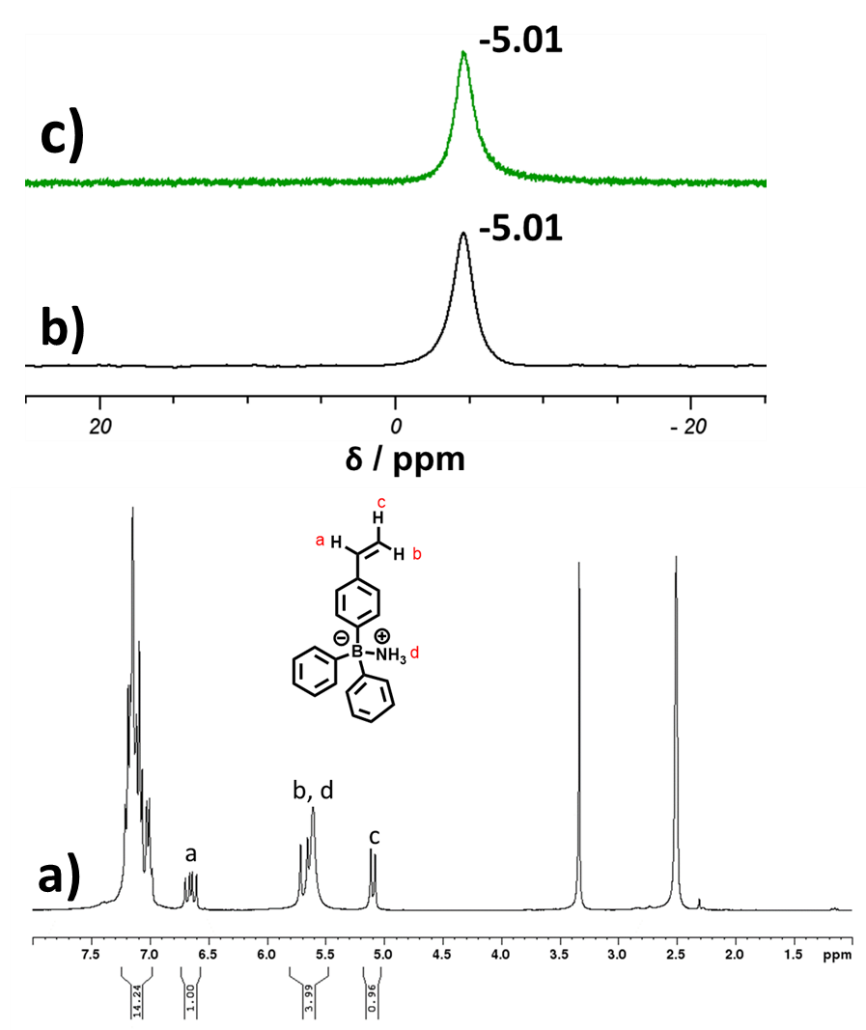


Figure SI 1: ^1^H and ^11^B{^1^H} NMR spectra of 4-styryl-diphenylborane ammoniate in DMSO-d_6_. (a) ^1^H NMR spectrum showing characteristic proton signals (b) ^11^B{^1^H} NMR spectra recorded immediately after synthesis and after prolonged storage, demonstrating the stability of the boron environment over time.


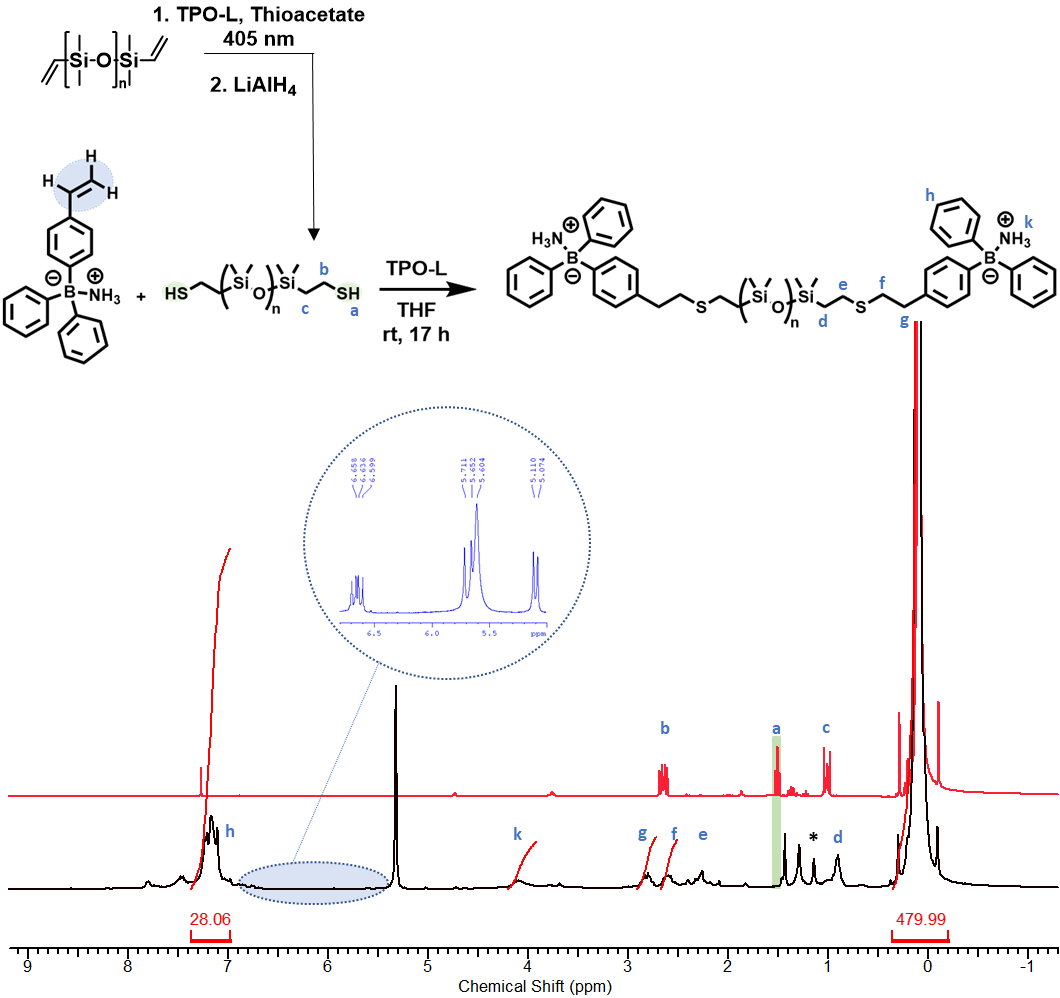


Figure SI 2: **¹H NMR spectra in** CD_2_Cl_2_ **confirming the functionalization of divinyl-terminated PDMS via thiol-ene addition and subsequent reduction to α,ω-dithiol PDMS. Characteristic peaks corresponding to the thiol and vinyl groups disappear after the reaction, indicating full conversion.**


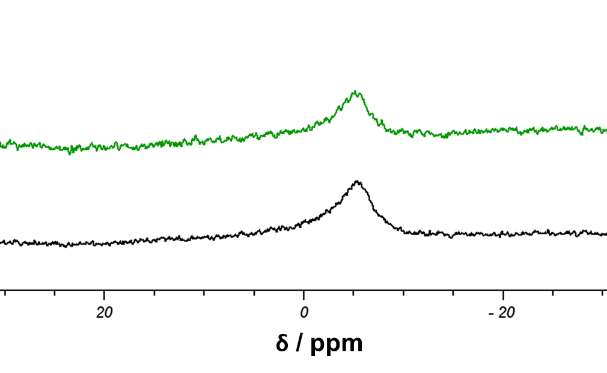


Figure SI 3: ^11^B NMR spectra of organoborane α,ω-chain end-functionalized **PDMS 1** in CD_2_Cl_2_, demonstrating its stability over time. No significant changes in the boron signal indicate that the material remains stable and resistant to hydrolysis under ambient conditions.


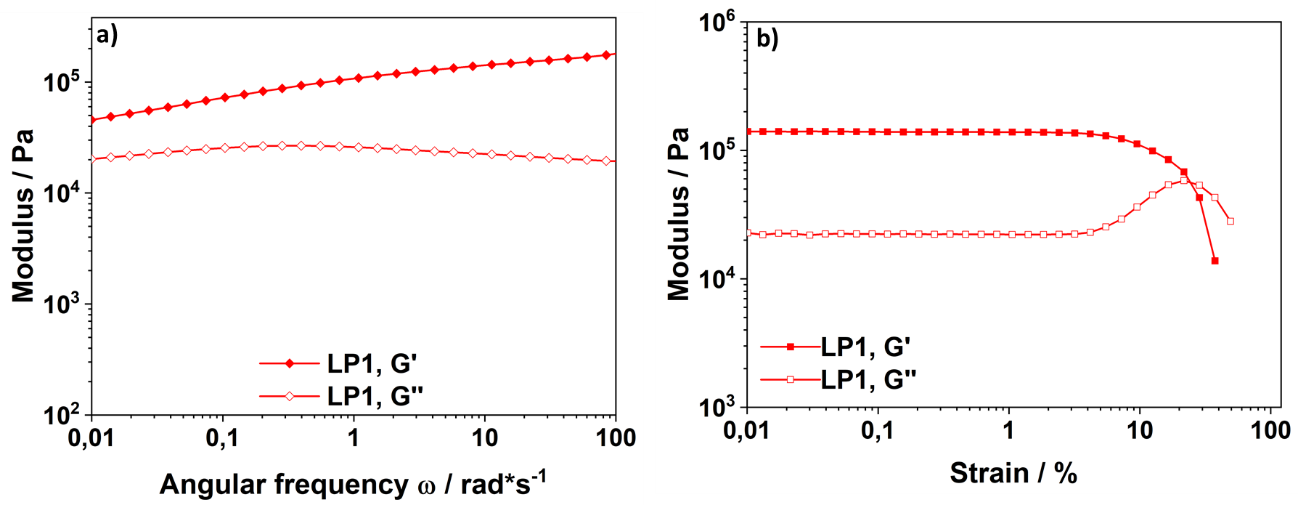


Figure SI 4: **a)** Frequency sweep of LP1 showing an elastic-dominated response with G′>G′′. **b)** Amplitude sweep of LP1.


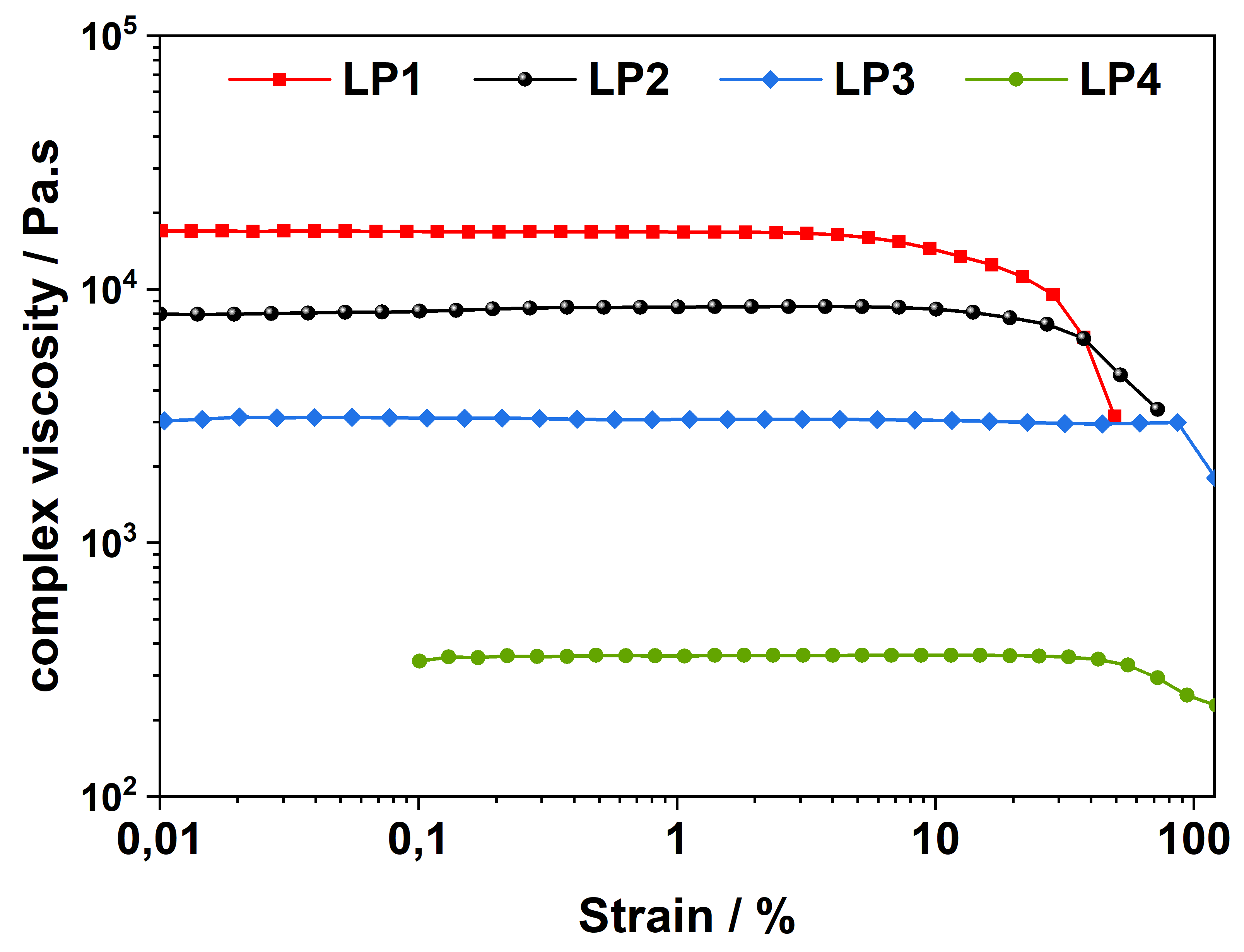


Figure SI 5: Amplitude sweep rheology of LP networks. The complex viscosity (η^∗^) is plotted as a function of strain. The data illustrate the strain-dependent viscoelastic behavior of the dynamically crosslinked networks, with LP1 exhibiting the highest viscosity and strain resistance, while LP4 demonstrates the lowest viscosity, indicating a more fluid-like behavior under deformation.


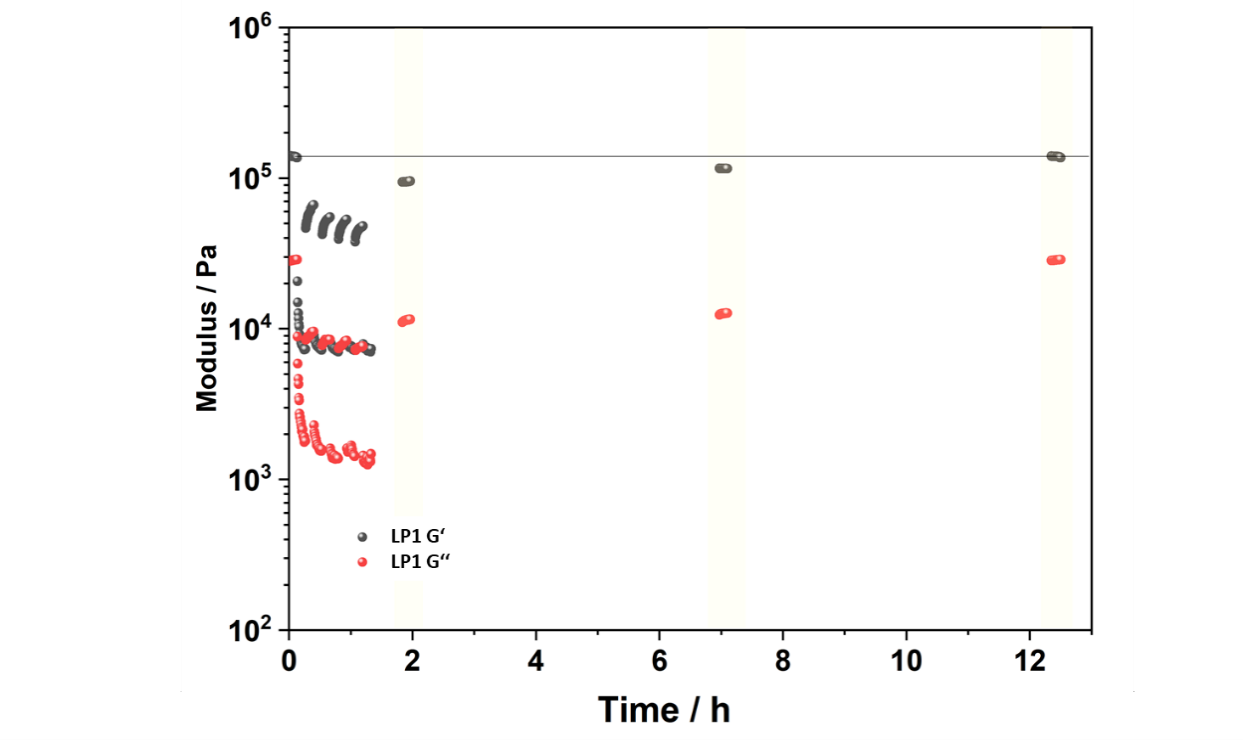


Figure SI 6: Time-dependent oscillatory rheology measurements of LP1 assessing the healing efficiency. After about 13 hours, the material exhibited nearly complete reformation, restoring up to 92% of its original mechanical properties.

Table SI-1 summarizes the different formulations used in this study, showing the variation in the ratio of the Lewis bases P4VP and AMS162. These formulations were selected to investigate the effect of LB composition on network dynamics, mechanical properties, and self-healing behavior.

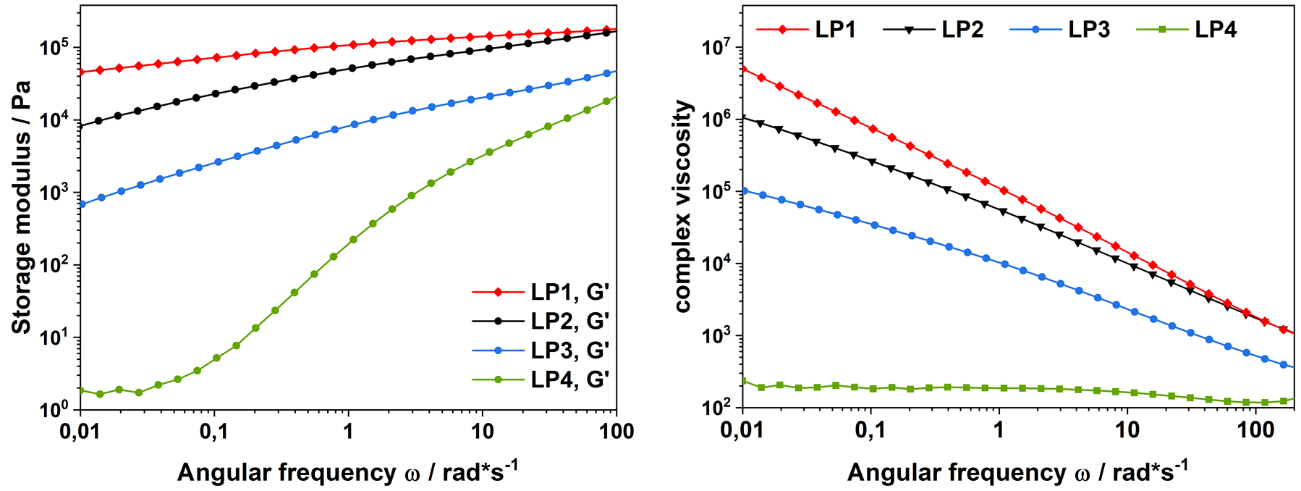


Figure SI 7, left: Storage modulus (G′) as a function of angular frequency, demonstrating the viscoelastic behavior of the dynamically crosslinked networks. Right: Complex viscosity (η^∗^) as a function of angular frequency, showing shear-thinning behavior characteristic of reversible crosslinked polymer systems. Variations in modulus and viscosity values highlight differences in network strength and relaxation dynamics.


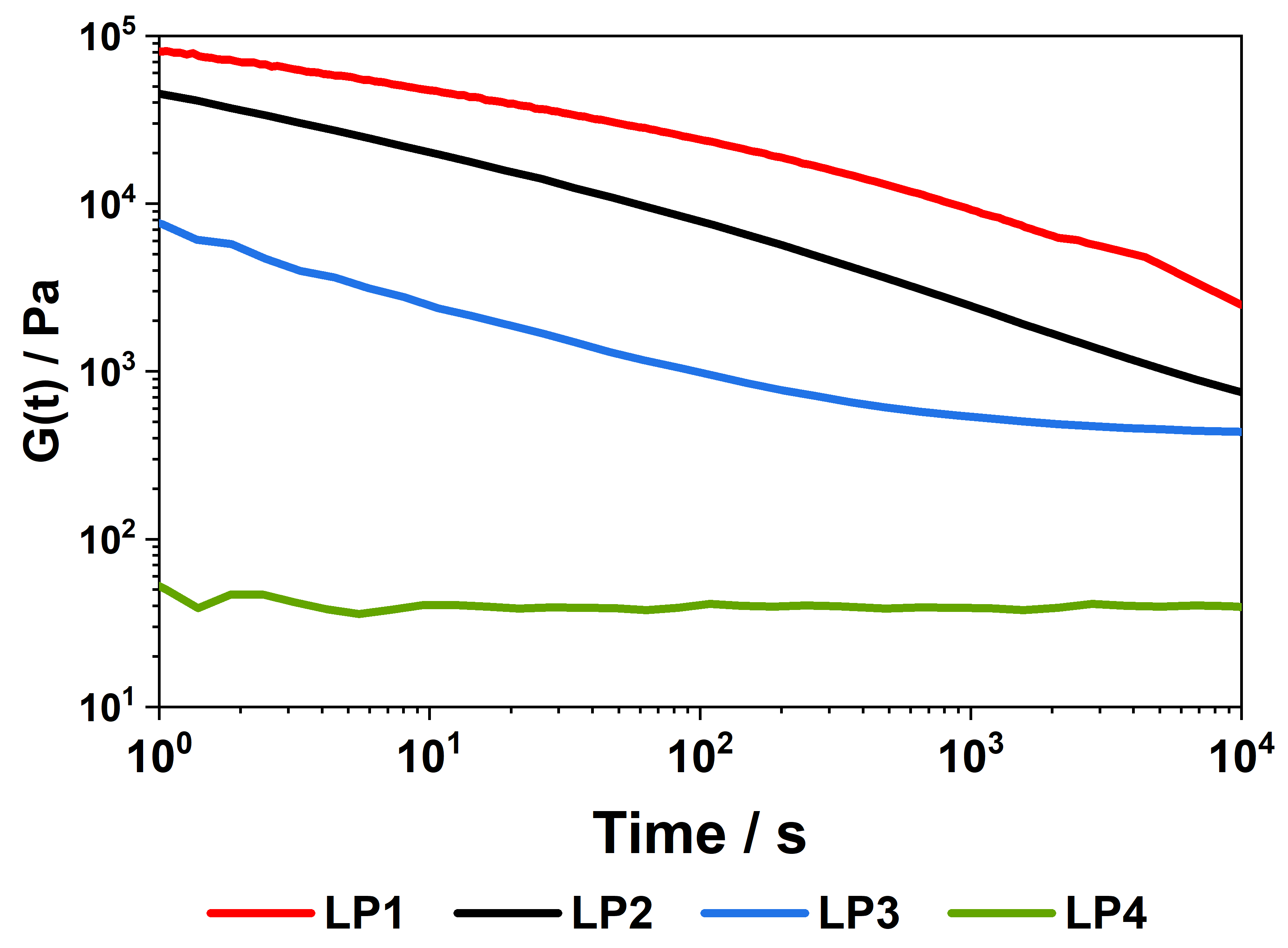


Figure SI 8: Complementary to the Frequency Sweeps, Stress Relaxation Tests were performed exemplarily for dedicated samples at room temperature. To remain within the linear viscoelastic range (LVE), a strain of 1% was used uniformly.


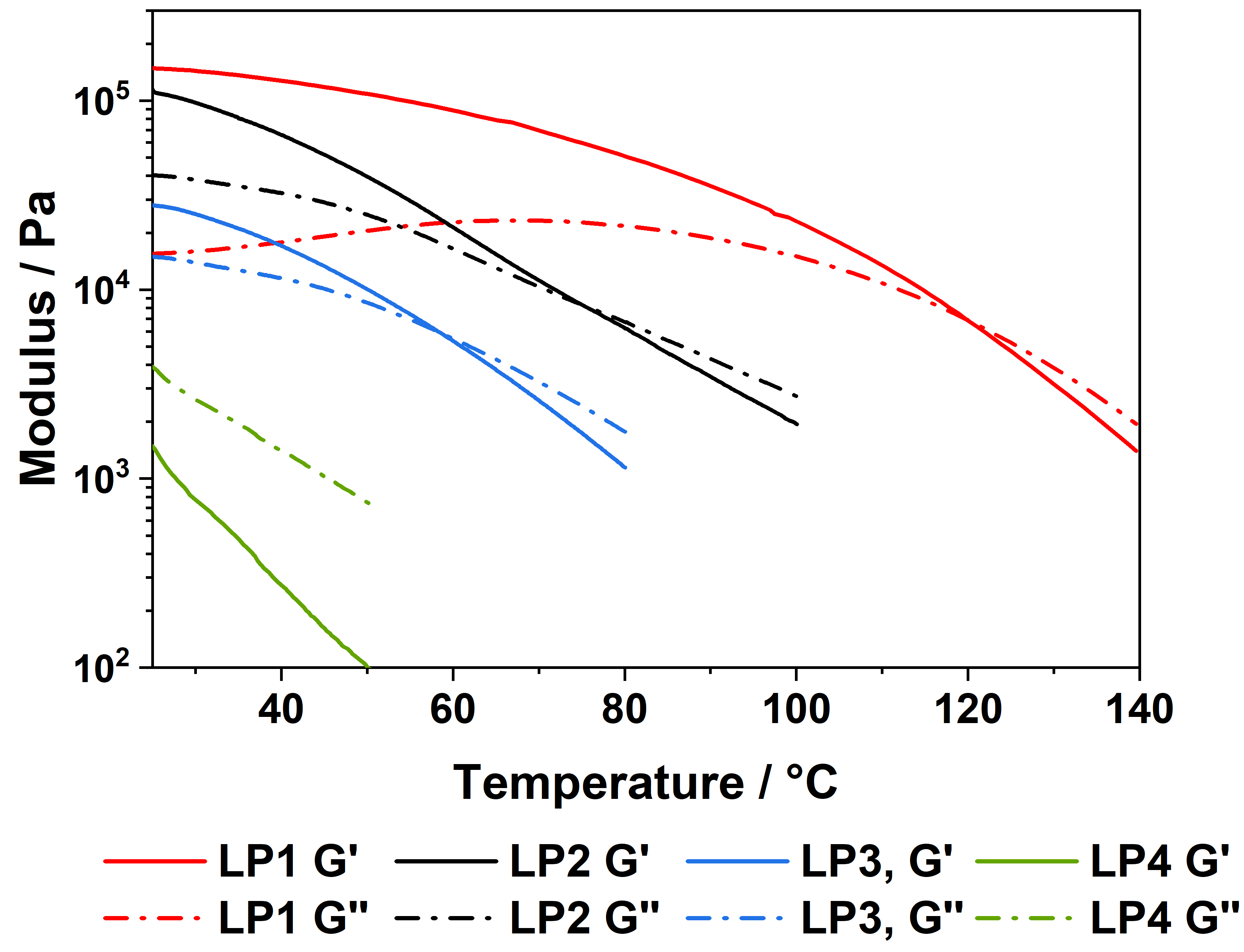


Figure SI 9: Temperature-dependent storage and loss moduli (G′, G′′) of all formulations LP1–LP4, measured by oscillatory rheology. LP4 shows a rapid decrease in G′ and low overall modulus values, indicating weak structural integrity and dominant viscous behavior at elevated temperatures. Data complement Figure 7a in the main text.


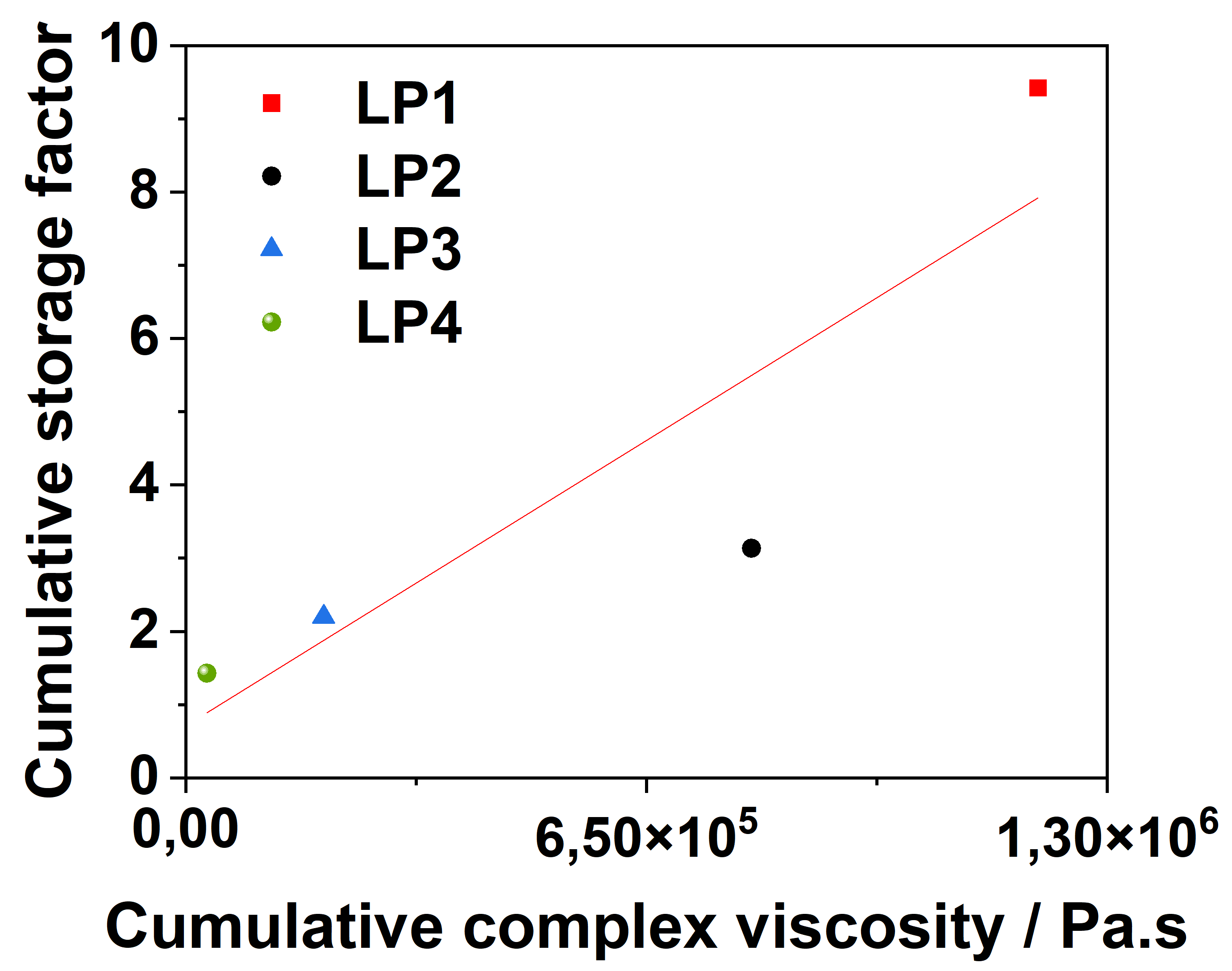


Figure SI 10: Cumulative storage factor (CSF) plotted against cumulative complex viscosity for LP1–LP4. The CSF represents the total elastic energy stored over the measured frequency range. The data confirm the correlation between network elasticity and viscosity, with LP1 showing both the highest CSF and viscosity, indicative of a robustly crosslinked and elastically dominant network.


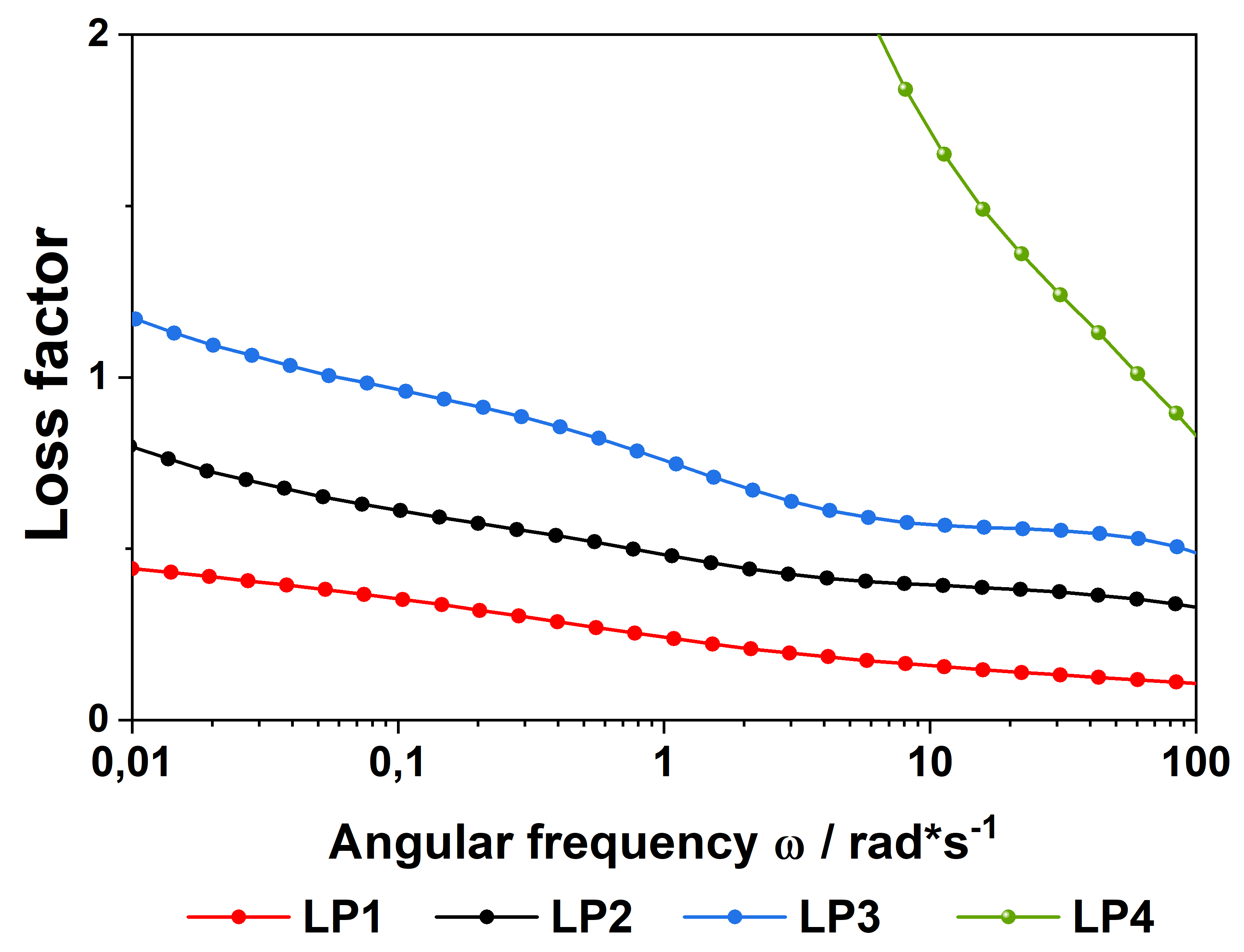


Figure SI 11: Frequency-dependent loss factor (tan δ) of LP1–LP4, measured via oscillatory shear rheology.


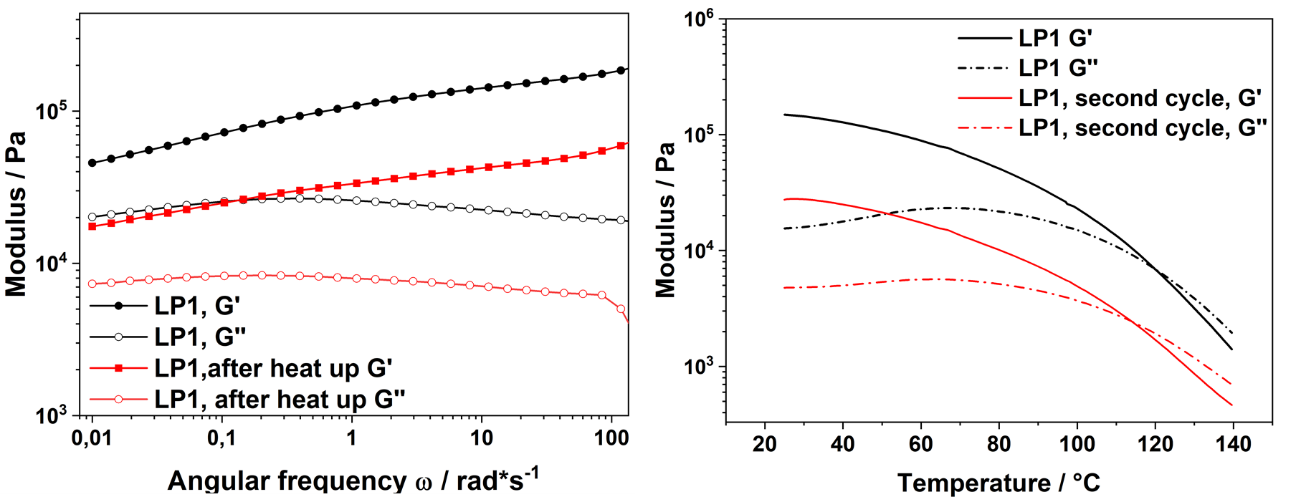


Figure SI 12: Thermal stability and reprocessing behavior of LP1. Left: Frequency sweep rheology before and after heating, demonstrating partial recovery of the storage (G′) and loss modulus (G′′) after thermal treatment. Right: Temperature-dependent rheology of LP1 over two heating cycles, showing the influence of temperature on the modulus and network integrity.


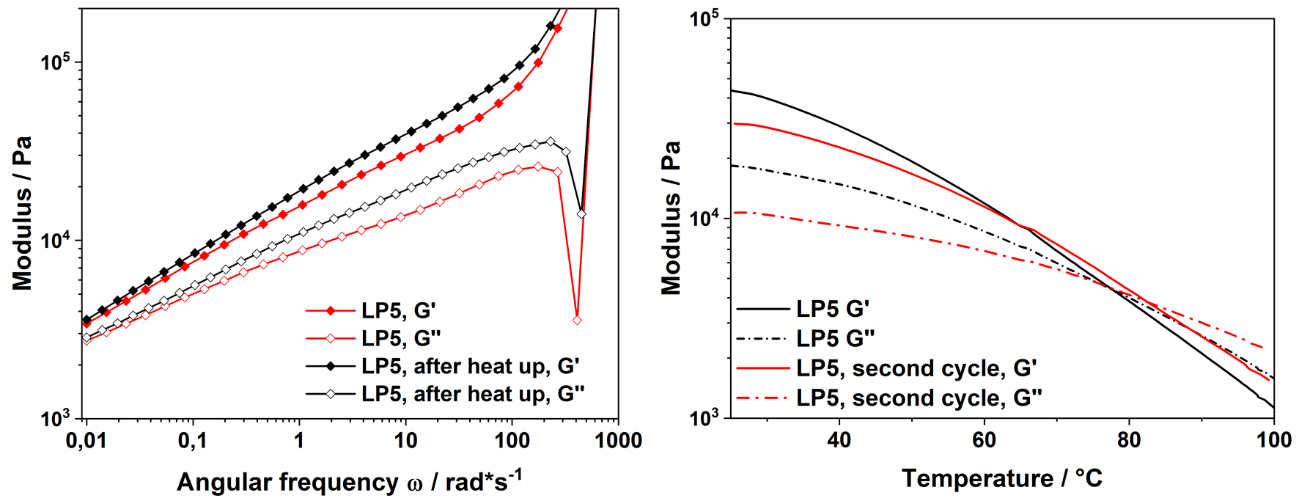


*Figure SI 13: Thermal stability and reprocessing behavior of LP5. Left: Frequency sweep before and after thermal treatment shows nearly complete recovery of storage (G′) and loss modulus (G″), indicating effective reformation of the dynamic network. Right: Temperature-dependent rheology over two heating cycles confirms good thermal responsiveness and reversible modulation of viscoelastic properties.*
